# Clinical and magnetic resonance imaging outcome after proximal hamstring tendon repair at mean 3 years follow-up

**DOI:** 10.1007/s00402-024-05684-w

**Published:** 2025-01-15

**Authors:** Carlo Sgustav, Lucca Lacheta, Ulrich Stöckle, Doruk Akgün, Dominik Geisel, Hi-Un Park, Adrian Marth, Suchung Kim

**Affiliations:** 1 Center for Musculoskeletal Surgery, Charité Universitätsmedizin Berlin, Augustenburger Platz 1, 13353 Berlin, Germany; 2 Department for Radiology, Campus Virchow Klinikum, Charité Universitätsmedizin Berlin, Augustenburger Platz 1, 13353 Berlin, Germany; 3 Department of Orthopaedic Sports Medicine, Technical University of Munich, Ismaninger Straße 22, 81675 Munich, Germany; 4 Klinik Für Orthopädie Und Unfallchirurgie, Martin Luther Krankenhaus Berlin, Grunewald, Caspar Theyß Strasse 27-31, 14193 Berlin, Germany; 5 DocOrtho MVZ Berlin, Friedrichstraße 94, 10117 Berlin, Germany

**Keywords:** Hamstring muscles, Proximal hamstrings tendon rupture, Proximal hamstrings tendon repair, Clinical outcome, Isometric strength, Magnetic resonance imaging (MRI), Fatty infiltration, Muscle volumetry, Muscle atrophy

## Abstract

**Purpose:**

The purpose of this study was to assess clinical and radiological outcome in patients after proximal hamstring tendon repair. We hypothesized that there is a significant correlation among subjective clinical outcome and interlimb asymmetries in muscle strength, fatty infiltration, and hamstring volume.

**Methods:**

This retrospective monocentric case series included patients with surgical repair after proximal hamstring tendon rupture. Clinical outcome was assessed utilizing: Healthy Days Core Module (CDC HRQOL-4), numeric pain rating scale (NRS), modified Harris Hip Score (mHHS), Tegner Activity Scale (TAS), return to pre-injury activity level (RTPA), and patient satisfaction score. Postoperative hamstring strength was measured using a handheld dynamometer and radiological outcome was determined by postoperative magnetic resonance imaging (MRI).

**Results:**

Twenty-seven patients with a mean age of 51.2 (± 12.6) years were available for follow-up at a mean of 41.11 (± 18.4) months. Patients state a mean of 10.6 (± 11.5) days in the unhealthy days (UHD) index and 88.9% show “good health” in the simple summary score (SSS). Mean subjective outcome scores were as follows: NRS 1.1 (± 2.4), mHHS 90.3 (± 14.8) and TAS 5.7 (± 2.2). A total of 59.3% RTPA and 88.9% state to be somewhat or very satisfied with their surgery. Mean interlimb strength ratio was 0.88 (± 0.21). MRI demonstrated a fully restored muscle–tendon unit, significantly greater fatty infiltration in the injured hamstrings (*p* = 0.009, *d* = 0.558), and a mean interlimb hamstring volume ratio of 0.94 (± 0.11). With respect to the 10% benchmark, patients had no significant asymmetries in muscle strength (*p* = 0.677, *d* = 0.084) or hamstring volume (*p* = 0.102, *d* = − 0.34). Correlation analysis revealed moderate correlation among asymmetries in strength and volume (*p* = 0.073, *r* = 0.373). In patients with the operated side inferior to the healthy side (*n* = 15), there was strong correlation among asymmetries in strength and volume (*p* = 0.002, *r* = 0.725). Statistically significant correlation was found between interlimb muscle volume atrophy and increase in fatty infiltration (*p* = 0.015, *r* = 0.481).

**Conclusion:**

Proximal hamstring repair results in good clinical outcome with satisfactory recovery of hamstring strength and volume. Interlimb asymmetries, in terms of muscle strength, fatty infiltration, and hamstring volume do not correlate with clinical outcome.

**Study Type:**

Retrospective cohort study; Level of evidence, 3.

## Introduction

Hamstring muscle injuries are common in professional athletes performing high-risk sports such as soccer, sprinting or water skiing. They make up 37% of all muscle injuries in professional soccer athletes and 50% of the muscle injuries in sprinters [13, 51]. Hamstring injuries become more frequent in middle-aged patients, as they continue to participate in sports and diagnostics become better established [7]. The typical mechanism of injury is an eccentric contraction due to a forced hyperflexion of the hip combined with knee extension [7, 50]. Injury of the hamstrings is most commonly found at the musculotendinous junction, but can occur anywhere between the muscle’s origin and insertion, allowing for various pathologies that range from harmless muscle strains to avulsions of the proximal hamstring origin [8, 10, 22, 27]. Magnetic Resonance Imaging (MRI) is the gold standard in diagnostics of proximal hamstring injury and should be performed whenever an injury is suspected [2, 27]. It provides excellent contrast in soft tissue with high spatial resolution and easily demonstrates the full extent of the injury [16, 27]. The injured side should always be compared to the non-injured to note and compare marginal differences. Surgical treatment is recommended for osseous avulsion fracture injuries with > 2 cm dislocation, proximal tendon avulsions of all three hamstring tendons, and proximal hamstring avulsions of two tendons with > 2 cm retraction [4, 7, 28]. A systematic review of 18 studies and 298 patients found that surgical therapy resulted in significantly better outcome with regard to patient satisfaction, return to sport, hamstring strength, and complication rates [17].

Previous studies used non-validated subjective clinical variables such as pain (NRS), return to pre-injury activity level (RTPA), patient satisfaction, modified Harris Hip Score (mHHS), and Tegner Activity Scale (TAS), as well as isokinetic strength testing to evaluate the postoperative outcome after proximal hamstring avulsion [3, 37, 46]. For strength testing, a restored muscle strength of ≥ 90% compared to the healthy limb is considered satisfactory, as an asymmetry of < 10% is considered “normal” in a healthy population and patients are expected to perform functionally equivalent to uninjured individuals [39]. Only a few studies performed MRI at follow-up and assessed radiological outcome in terms of tendon healing, fatty infiltration, muscle atrophy, and tendinopathy [5, 24, 25, 31, 33, 34, 40, 41, 45]. To our knowledge, only one previous study investigated postoperative muscle volume of the hamstrings [34]. MRI serves as the gold standard for the evaluation of muscle volume (MV) assessment by three-dimensional segmentation, which can be done manually slice-by-slice with good results in terms of inter- and intraobserver variability [35, 42]. The literature and clinical benchmark of 10% also applies to the evaluation of muscle volume asymmetries. Kulas et al. reported a mean relative hamstring muscle volume asymmetry of 2.8% in a cohort of healthy individuals, so that muscle volume in uninjured individuals can be assumed to be nearly symmetrical [23].

The objective of this study was to assess clinical outcome, muscle strength, and radiological degeneration in terms of fatty infiltration and muscle volume atrophy after proximal hamstring tendon repair, to investigate if there is significant correlation among these findings. We expected patients to show excellent results in terms of clinical functional outcome and tendon–muscle quality along with satisfying recovery of hamstring strength and volume of the injured leg. Furthermore, we hypothesized that there would be significant correlation among clinical outcome and interlimb asymmetries in terms of muscle strength, fatty infiltration, and hamstring volume.

## Material and methods

This monocentric retrospective study was designed for patients after proximal hamstring tendon repair to verify satisfactory clinical and functional rehabilitation.

### Patient selection and data collection

Included were patients with rupture of at least one proximal hamstring tendon from 2015 to 2020 that subsequently underwent surgical repair. Diagnosis was based on clinical examination and hereafter confirmed by MRI. Indication for operative treatment was given in cases of persisting pain and weakness, clinical signs of retraction, and MRI-confirmed tendon ruptures. Patients that met the inclusion criteria were identified and contacted. An appointment was arranged at our institution to complete the questionnaire, perform bilateral isometric strength testing, and undergo MRI of the hip and upper thigh. Information on surgery, rehabilitation and demographic data were obtained from the patient’s medical record. Patients with bilateral proximal hamstring ruptures, advanced Coxarthrosis, re-ruptures, or polytraumatic injuries were excluded.

All participants gave their informed and written consent and were thoroughly informed about the procedures in advance. The study was approved by the institutional review board (EA1/374/20).

### Surgical technique

Surgical procedure has already been described thoroughly in several publications, thus only the most relevant steps are listed [7, 21, 32]. All patients were operated at a single institution by two experienced orthopedic surgeons and received the identical surgical procedure. Patients were positioned in prone position on the operating table and received general anesthesia. A transverse skin incision of 5-8 cm in the gluteal fold was used for operational access. Attention was paid to not injure the posterior femoral cutaneous nerve. Subcutaneous fatty tissue and superficial fascia were severed and retracted, and the gluteus maximus muscle elevated superiorly. The hamstring fascia was opened, and in cases of acute rupture hematoma was emptied out. In cases of chronic ruptures, scar tissue was carefully dissected and removed. Then the sciatic nerve was identified, released from perineural adhesions, and thereafter protected throughout the remaining operation. The proximal tendon stump was identified, and residual tissue was removed. To secure optimal reattachment, the ischial tuberosity was decorticated with a curette, and up to three titanium corkscrew anchors (Arthrex, Naples USA) were used to reattach the tendon to its footprint at the refreshed bone. The sutures were passed through the tendon stump using a free needle and were tied on top of the reattached tendon.

### Rehabilitation protocol

All patients received a standardized early functional, and partly orthosis-free aftercare (Table 1). Physiotherapy started on the first postoperative day and patients gradually increased hamstring load under supervision. Progress follow-up and removement of the sutures was scheduled at 2 weeks postoperative.

**Table 1:** Aftercare scheme

*Rehabilitation protocol: Proximal Hamstring Repair*				
15 kg partial weight bearing on forearm crutches for a total of 6 weeks, small stride length, no forced hip flexion and combined knee extension, no passive stretching of hamstring muscles for a total of 3 months				
	Range of motion Splint treatment	Loading	Exercise program	Specifics
1. Day	NO active flexion in the hip joint over 60° with combined knee extension	15 kg partial weight bearing on 2 forearm crutches (3-point walking), short stride length	Thrombosis prophylaxis (active muscle pump) Lymphatic drainage Cryotherapy (Ice) Electrotherapy (TENS Quadriceps) Gait school (3-point-walking), Stairs	No passive stretching for a total of 3 Months Compression stockings (Class I)
2. – 4. Week			Isometric strengthening Lumbar-Pelvic-Hip region Lymphatic drainage Electrotherapy (TENS Quadriceps)	
5. Week			Isometric strengthening Lumbar-Pelvic-Hip region (PNF) Hip flexion up to 90° with equal knee flexion, extension free	Closed chain and short lever
From 6. Week	Active hip flexion and knee extension up to 90° with „dragging “ heel in SP	Start of full load (free walking)	Free walking without forearm crutches Active hip flexion and knee extension in SP and assistive in PP Isometric activation of hamstring muscles in SP	Active pelvic mobilization Further load increase ONLY after release
From 8. Week			Isokinetic strengthening in closed chain (leg press with body weight) Aquatraining Crosstrainer Bicycle ergometer Start eccentric training hamstrings in SP	Closed chain and short lever
From 4. Month			Easy jogging possible	Increase pain free
From 5. Month			Sports specific training	

^*^TENS Transcutaneous Electrical Nerve Stimulation, *PNF* Proprioceptive Neuromuscular Facilitation, *SP* supine position, *PP* prone position

### Questionnaire

The follow-up survey encompassed clinical subjective scores and questions concerning demographic data, patient satisfaction, return to pre-injury activity level, and postoperative complications. Pain was evaluated by the pain numeric rating scale (NRS). Subjective hip function was assessed with the modified Harris Hip Score (mHHS), and the level of athletic activity was determined by the Tegner Activity Scale (TAS). Patient satisfaction was assessed using a patient satisfaction score questionnaire that consists of three questions with each five-point response scales ranging from 5 “very satisfied” to 1 “very dissatisfied”. Similar measures have been used in the literature and proved to be reliable and valid [15, 19, 38, 47]. Health-related quality of life was assessed by the Healthy Days Core Module (CDC HRQOL-4) and two summary indices were generated: the unhealthy days (UHD) index and the simple summary score (SSS) [12]. The UHD-index is the sum of the physically and mentally unhealthy days, capped at 30 total days. The SSS features all four items of the HRQOL-4, which are dichotomized and summed to create the final score. Self-rated general health status was evaluated as a solitary measure of HRQOL [12].

### Isometric hamstring strength testing

Bilateral isometric strength testing was performed with a handheld dynamometer (IsoForceControl® EVO2, Oberburg, Switzerland). All patients were tested with the same dynamometer, in a standardized setting, and received the same directions. Patients laid down in prone position with the dynamometer fixed to a wall at the foot of the examination bed. It was positioned at the level of the patient’s ankle with the knee bent 90° so that the lower leg was perpendicular to the examination bed. Patients were directed to remain stationary and not lift their hips during testing. They were then instructed to pull their heel toward their buttocks. Testing was performed unilaterally, alternating between the operated and contralateral limb. Two rounds per leg were executed, so that in total four measurements could be taken. Relevant for statistical analysis was the respective maximal force achieved on the second run. The force of the injured limb was compared to the contralateral limb, and a percentage difference, along with the limb symmetry index (LSI) were calculated. A side–to–side difference of less than 10% (LSI > 90%) was considered non-pathological.

### Radiological examination and MRI protocol

**Fig. 1:**
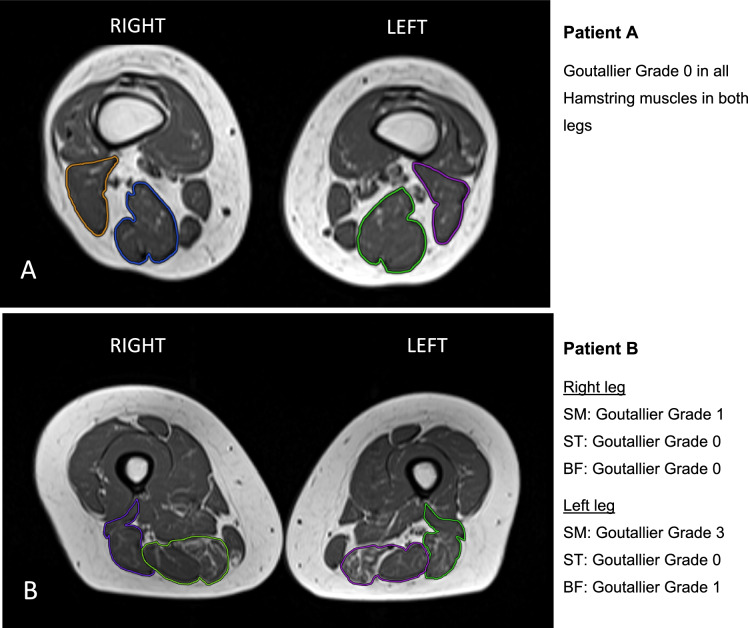
Goutallier grading of two different patients (**A** and **B**)

**Fig. 2:**
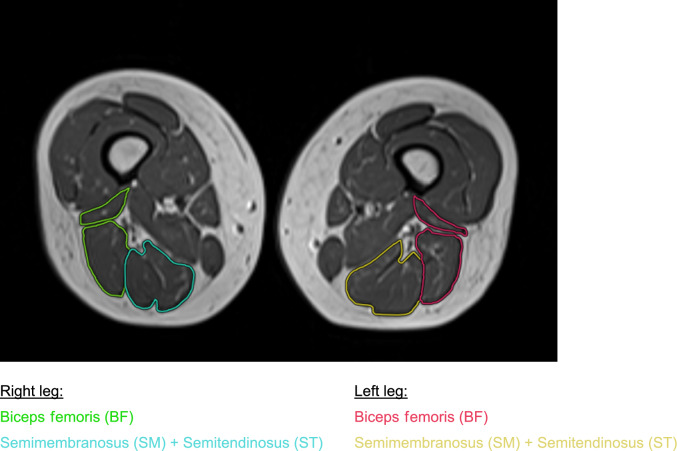
Quantification of muscle volume (MV) using manual slice-by-slice segmentation

Images of the pelvis and thigh were acquired on a 1.5 Tesla scanner (MAGNETOM Avanto, Siemens Healthineers, Erlangen, Germany) with patients in a supine position by using a multichannel phased-array coil. Three sequences were used to cover the examination area from pelvic girdle to femur, femur to knee, and from knee to proximal tibia with a 0.5 cm intersection gap. The protocol consisted of the following: (1) T2-weighted axial turbo inversion recovery magnitude (TIRM), Time to repeat (TR) 6000 ms, Time to echo (TE) 43 ms, slice thickness (ST) 4 mm, field of view (FOV) 400 × 319 mm2, matrix size 408 × 512 (2) T1-weighted axial images, TR 600 ms, TE 10 ms, ST 4 mm, FOV 400 × 320 mm2, matrix size 256 × 320 (3) coronal TIRM, TR 5300 ms, TE 43 ms, ST 3 mm, FOV 450 × 450 mm2, matrix size 512 × 512 (4) sagittal proton-density (PD) weighted turbo spin echo (TSE) images, TR 3700 ms, TE 43 ms, ST 3 mm, FOV 400 × 400 mm2, matrix size 256 × 256. Image analysis was performed by an institutional radiologist with four years of experience in musculoskeletal imaging using Visage Imaging Client (Software Release v7.1, Visage Imaging, Berlin, Germany). TIRM and PD-weighted sequences were used for qualitative assessment of the examination area. Semiquantitative analysis of fatty muscle infiltration was performed by adaption of the Goutallier classification [14], which is a semiquantitative scoring system, initially developed for the determination of fatty muscle infiltration of the rotator cuff muscles in computed tomography (CT), which is also applied to MRI in current clinical practices (Fig. 1) [14, 26, 45]. The Goutallier classification consists of the following: Grade 0 = no fat visible, Grade 1 = fatty streaks visible, Grade 2 = more muscle than fat visible, Grade 3 = muscle and fat equally visible, Grade 4 = more fat than muscle visible. Manual slice-by-slice segmentation for muscle volumetry was performed in T1-weighted images by seeding regions of interest (ROI) in the semintendinosus (ST), semimembranosus (SM) and the short and long head of the biceps femoris (BF) muscle from origin to insertion (Fig. 2).

### Statistical analysis

All Data was analyzed using IBM SPSS Statistics Version 28.0.1.0 for Windows. Demographic data and results of the clinical outcome scores are presented as a mean (± SD). Patient satisfaction, SSS, RTPA, postoperative complications, muscle strength asymmetry (LSI), volume asymmetry, and qualitative muscle conspicuities are expressed as percentages or fractions. A *p*-value < 0.05 was considered as statistically significant. One-sample t-test analysis was used to determine the significance of interlimb asymmetry in muscle strength and hamstring volume. Due to the small sample size, the results of the t-tests were additionally checked with non-parametric methods to control for possible precondition violations of the parametric tests (e.g., Wilcoxon test). Wilcoxon signed rank test was also used to compare fatty infiltration (Goutallier scores) between the two legs. Unpaired t-test analysis was conducted to assess asymmetries in hamstring strength and volume between men and women. Cohens d was used as an effect measure for group differences: *d* = ± 0.2 small effect, *d* = ± 0.5 medium effect, *d* = ± 0.8 large effect [6]. Spearman correlation coefficient was performed to evaluate correlation among clinical outcome scores, strength measures, and radiological outcomes. Cutoff values for the effect size of correlations were set as follows: *r* = ± 0.5 strong relationship, *r* = ± 0.3 moderate relationship, *r* = ± 0.1 weak relationship [6]. Hypothesis testing was initially conducted with all enrolled patients and then repeated with the patients whose operated limb’s performance was inferior to the healthy one, as originally expected.

## Results

In total 27/34 patients (79.4%) with a mean age of 51.2 (± 12.6) years were available for follow-up at a mean of 41.11 (± 18.4) months. Two patients (5.9%) could not be contacted, two (5.9%) were unavailable for follow-up, and three (8.8%) did not wish to participate in the study. Demographic data of all included patients is presented in Table 2.

**Table 2:** Demographic Data

	Total (± SD)
Number of patients (N)	27
Age (years)	51.2 (± 12.6)
Sex (male/female)	12/15 (44.4/55.6%)
OP-Side (right/left)	10/17 (37.0/63.0%)
Time from injury to surgery (days)	48.7 (± 120.6)
Follow-Up (months)	41.1 (± 18.4)

^*^SD Standard deviation

The questionnaire was filled out completely by all patients. The mean subjective clinical outcome scores, return to pre-injury activity level, and patient satisfaction are shown in Table 3. Two patients experienced postoperative complications that required reoperation. One showed postoperative sensory deficits around the calf and slight muscle weakness in plantar flexion, the other had an abscess with a fistula tract in the hamstring area.

**Table 3:** Clinical outcome and return to pre-injury activity level

	Total (± SD)
UHD-index	10.6 (± 11.5)
SSS	
Good Health	24/27 (88.9%)
Poor Health	3/27 (11.1%)
NRS	1.1 (± 2.4)
mHHS	90.3 (± 14.8)
TAS	5.7 (± 2.2)
RTPA	16/27 (59.3%)
Patient Satisfaction Score	14.3 (± 1.5)

^*^SD Standard deviation, *UHD* Unhealthy days index, *SSS* Simple summary score, *NRS* Numeric rating scale, *mHHS* modified Harris Hip Score, *TAS* Tegner Activity Scale, *RTPA* Return to pre-injury activity level

At follow-up, 11.1% of the patients reported excellent, 25.9% very good, and 55.6% good health. Subjective clinical outcome in the NRS and mHHS was excellent and 59.3% of the participants have returned to their initial level of pre-injury activity level. Overall, 88.9% of the patients stated to be somewhat or very satisfied with their surgery and all patients (100%) declared that they would retrospectively choose surgery again as therapy.

Postoperative isometric strength testing was performed in 25/27 patients. Two patients could not be tested, because the Dynamometer was not available at follow-up. Mean maximal strength was 11 kg (± 6) on the injured and 13 kg (± 8) on the healthy side. Mean hamstring strength recovery was 88% (± 21%) on the operated side. A total of 72% of the patients had interlimb strength asymmetries greater than 10% (LSI < 90%). Nonetheless, with respect to the 10% clinical benchmark, one-sample t-test analysis and Wilcoxon-test revealed no significant strength asymmetries between the operated and contralateral limb (*p* = 0.677, *d* = 0.084). When performing subgroup analysis, men had greater interlimb strength asymmetry compared to female patients (m:18%, w:6%; *p* = 0.156, *d* = 0.586).

**Fig. 3:**
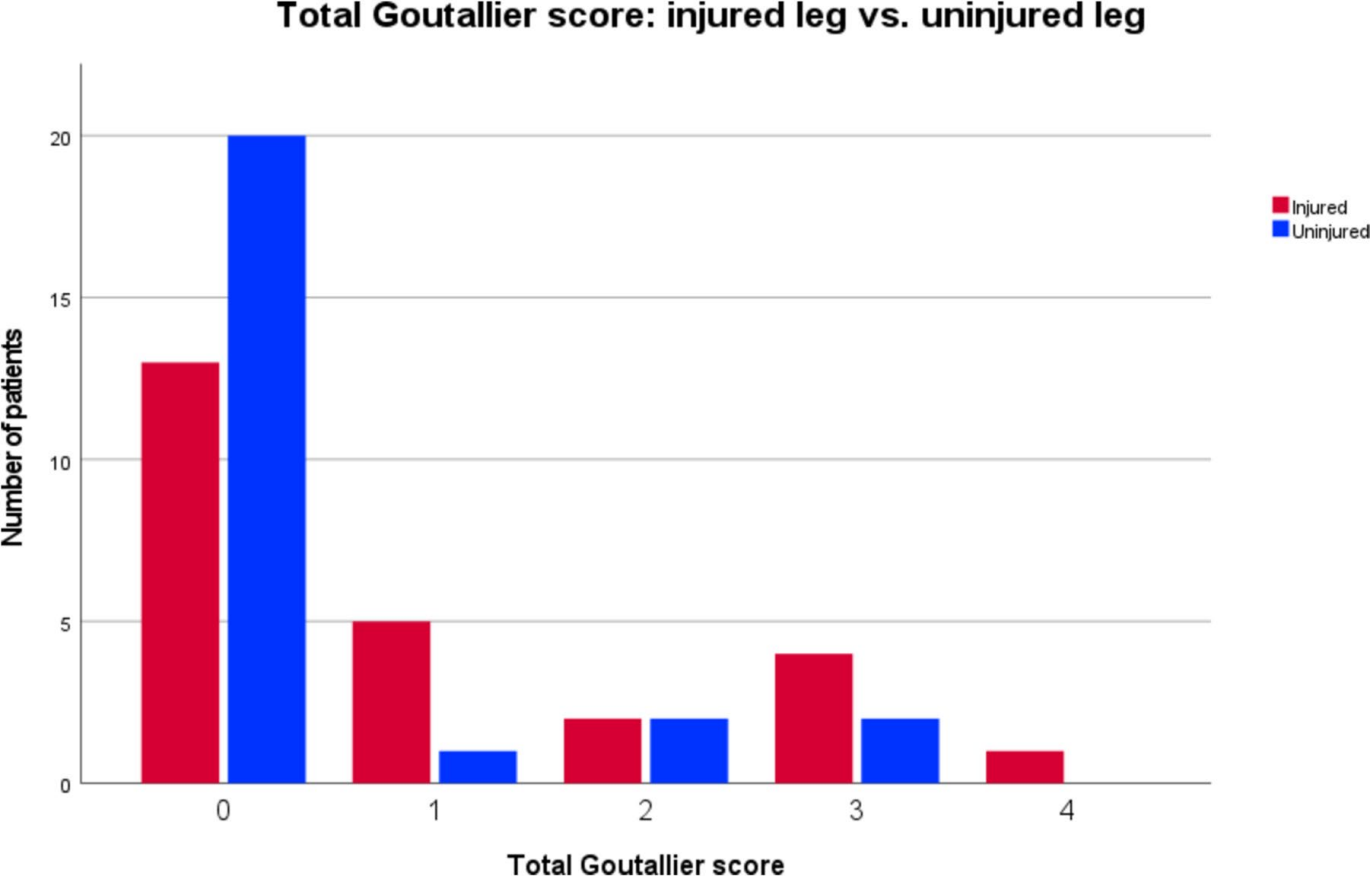
Distribution of total Goutallier score of injured and uninjured leg for all patients

**Fig. 4:**
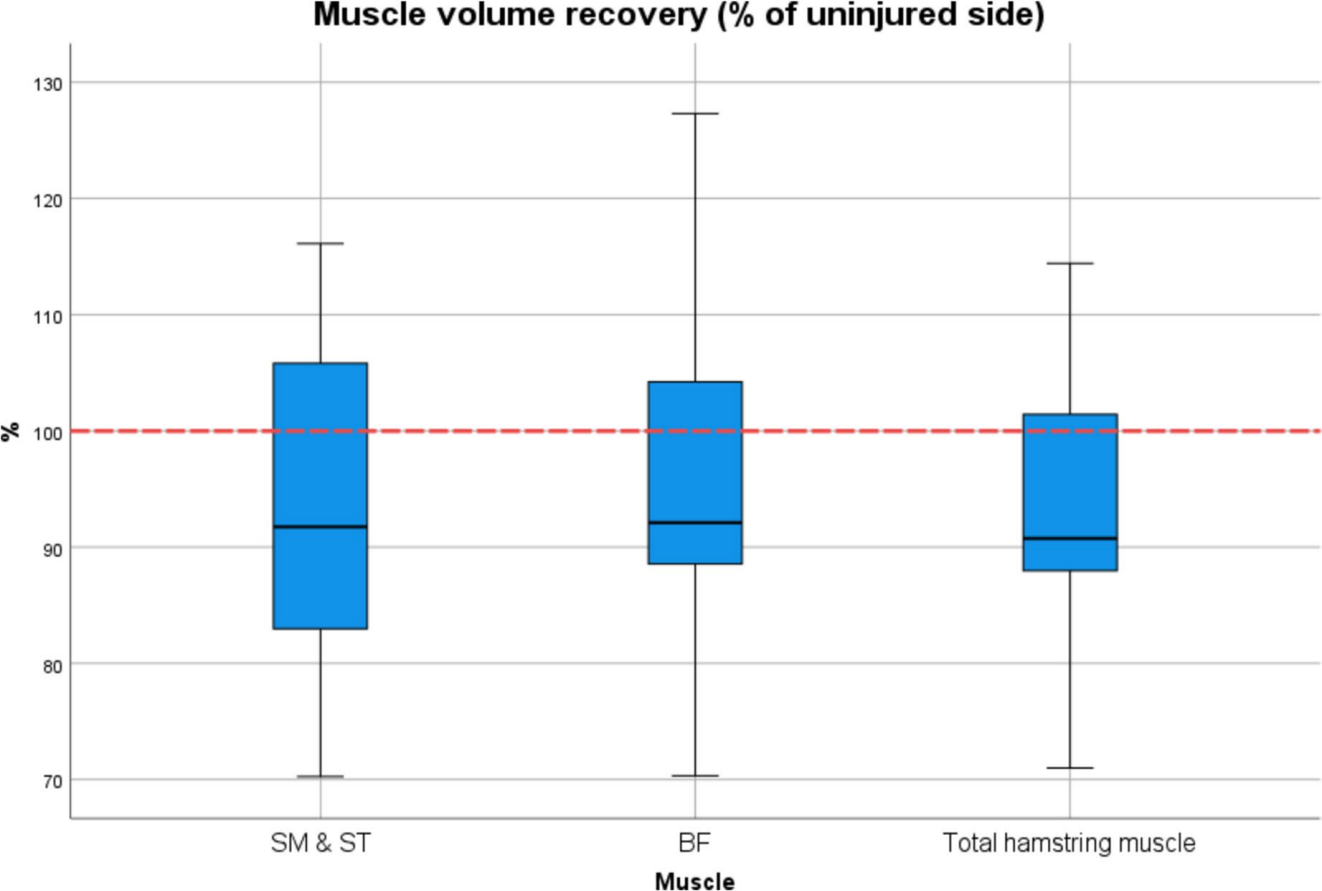
Recovered muscle volume of the injured leg (% of the uninjured side) of the semimembranosus (SM) & semitendinosus (ST), the biceps femoris (BF), and the total hamstring muscle

Radiological outcome was assessed in 25/27 patients. Two had to quit MRI examination due to claustrophobia. All patients showed an intact insertion of the tendon at the ischial tuberosity and a fully restored muscle–tendon unit. One patient had increased signal intensity changes in fluid-sensitive sequences in the obturator externus muscle adjacent to the repair site. Two patients (8%) demonstrated relevant fatty infiltration on the operated side (Goutallier grade 2 and 3), of which one also had infiltration (Goutallier grade 2) on the healthy side. In total, patients had significantly greater fatty infiltration (*p* = 0.009, *d* = 0.558) on the injured compared to the uninjured side (Fig. 3). Two patients had edema (8%) and one showed signs of tendinopathy (4%). Mean total muscle volume was 674 cm^3^ (± 228) on the injured and 735 cm^3^ (± 282) on the healthy limb with a mean hamstring volume recovery of 94% (± 11%) of the operated limb (Fig. 4). A volume deficit > 10% compared to the uninjured limb was found in 10/25 patients (40%). Considering the 10% benchmark, one-sample t-test analysis and Wilcoxon-test showed no significant muscle volume atrophy (*p* = 0.102, *d* = − 0.34). Men showed higher interlimb muscle volume atrophy opposed to women (m:10%, w:2.7%; *p* = 0.1, *d* = 0.687).

**Fig. 5:**
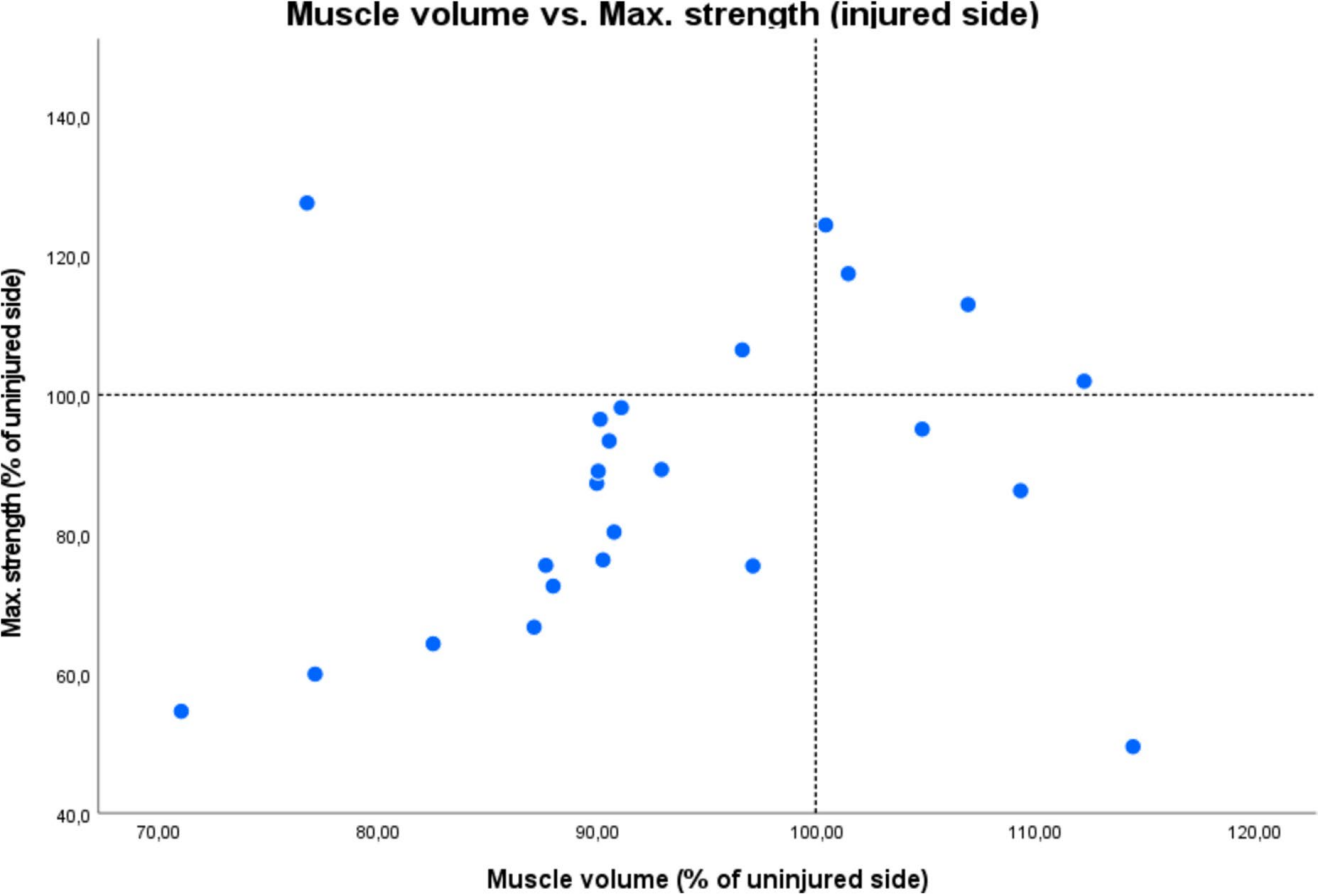
Scatterplot of muscle volume vs. max. strength of the injured side as a percentage of the uninjured. Reference lines illustrate the area where there is no difference between the two sides

**Fig. 6:**
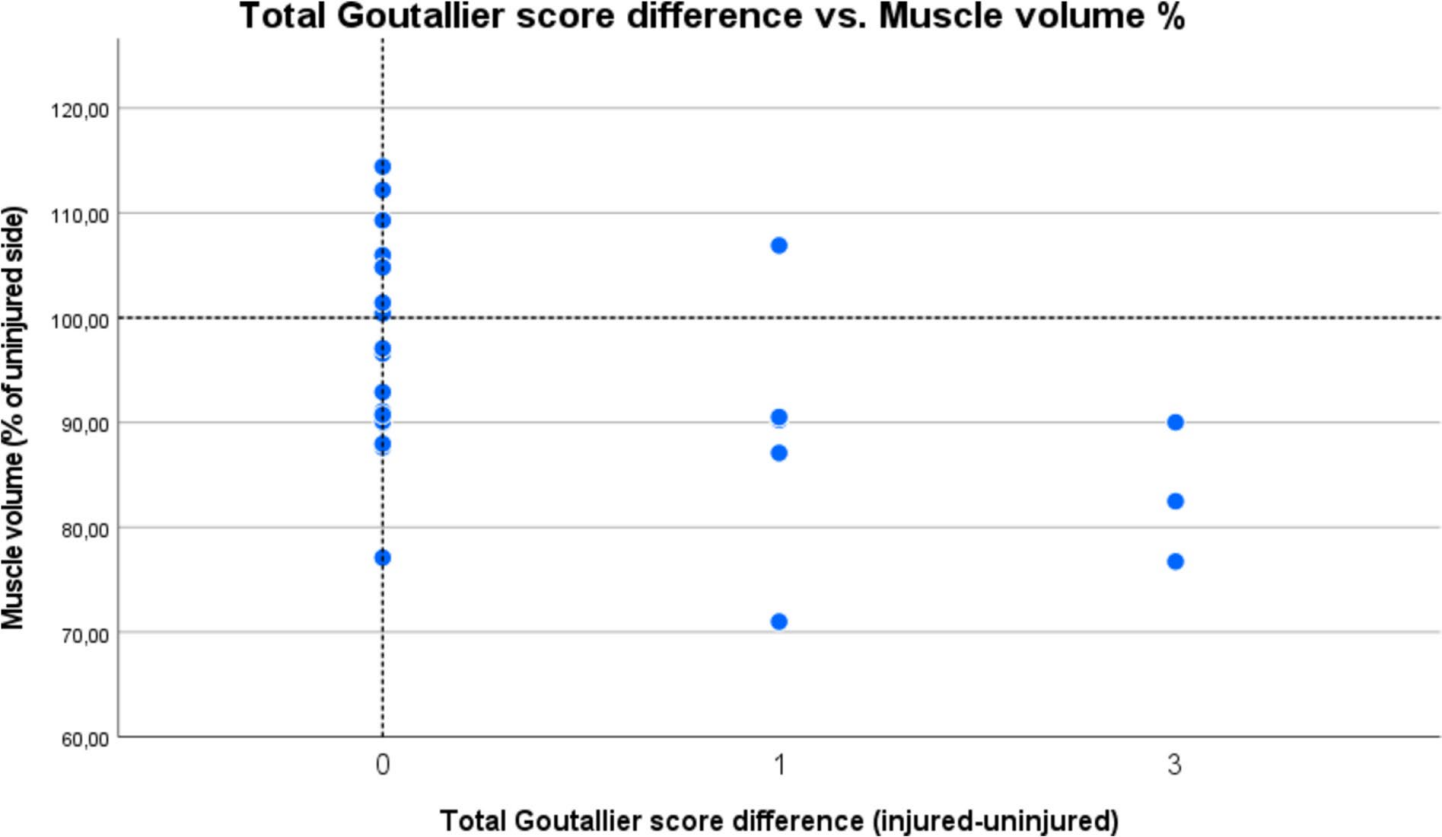
Scatterplot of interlimb Goutallier score difference vs. muscle volume (% of the uninjured side). Reference lines illustrate the area where there is no difference between the two sides

Correlation analysis revealed significant association between the examined functional outcome scores NRS, UHD-index, mHHS, and TAS (*p* < 0.05) and a significant correlation among postoperative muscle strength and volume (*p* ≤ 0.001). TAS significantly correlates with Total patient satisfaction, muscle strength, and muscle volume on both the operated and contralateral side (*p* < 0.05). There is a moderate correlation among strength asymmetries and volume asymmetries (*p* = 0.073, *r* = 0.373) (Fig. 5) and a strong association, when only patients with the operated limb inferior to the healthy limb (*n* = 15) are considered (*p* = 0.002, *r* = 0.725). Total Goutallier score difference between the injured and uninjured leg significantly correlates with interlimb muscle volume atrophy (*p* = 0.015, *r* = 0.481) (Fig. 6). Apart from the previously mentioned correlations, no further significant associations were found among the remaining clinical outcome scores, muscle strength, and muscle volume (n.s.).

## Discussion

The main finding of this study is that patients who underwent proximal hamstring tendon repair showed good clinical results with satisfactory recovery of hamstring strength, volume, and tendon integrity. Although good results were obtained both clinically and objectively, there are no significant correlations among subjective clinical outcome and interlimb asymmetries in muscle strength, fatty infiltration, and hamstring volume.

High satisfaction rates and good subjective clinical outcome coincide with the results of previous studies [3, 17, 20, 46]. Chahal et al. reported from 13 surgically treated patients with good outcome (HHS 90.7; VAS 1.3; TAS 4.6) at 36.9 months follow-up. Results concerning RTPA differ in literature and range from 55 to 100% [9, 46]. Willinger et al. found a return to pre-injury sports level of 49% [49]. In this study 16/27 patients (59.3%) resumed sports at their pre-injury level which lies within the range of the studies published to date.

According to a recent review, strength testing is only reported in 48.1% of the studies that evaluate outcome after proximal hamstring repair [37]. This is of great concern, as hamstring strength is predictive for recurrent muscle injuries and a major risk factor for lower limb joint injuries (e.g., anterior cruciate ligament) [1, 44]. In our study, patients had an average strength recovery of 88%. This supports the results of previous studies, where mean postoperative hamstring strength ranges from 78 to 101% compared with the contralateral leg [46]. A systematic review with 35 studies, observed a mean postoperative muscle strength of 87% in 460 patients at final follow-up [18]. Contrary to our expectations, some patients had more strength and greater muscle volume on the operated leg than on the uninjured. We believe that this is related to the dominance of the respective leg, which, however, we have not surveyed.

In our study, all patients show an intact insertion of the tendon at the ischial tuberosity and a fully restored muscle–tendon unit. This confirms that, to a certain degree, the applied surgical technique and the rehabilitation protocol were effective. A similar rate of tendon integrity and healing is described in literature. Pihl et al. observed 36 patients of which 29 (81%) showed successful healing and complete attachment of all hamstring tendons at MRI follow-up [34]. Excellent tendon healing rate was reported by Van der Made et al. [45] in 20/21 patients, with one patient having suffered re-injury due to a second water-skiing accident before the 1-year MRI control. Additional studies examined tendon integrity at follow-up (67 patients total) and all note a 100% successful healing rate [5, 24, 25, 31, 33, 40, 41]. In our study, one patient had increased signal intensity changes in fluid-sensitive sequences in the obturator externus muscle adjacent to the repair site. Increased signal intensity is considered to reflect increased intra- or extracellular water (muscle edema) which can be induced by inflammatory response by acute muscle injury or occur in the context of denervation edema [29, 48]. Overall, the cause of long-term high signal intensity changes in the skeletal muscle remains unclear, while low-signal intensity changes mostly represent newly developed fibrous tissue [11, 36]. In the present study, one can hypothesize that isolated signal intensity changes in one patient are not directly related to the repair, but rather to denervation processes or chronic peritendinous inflammation due to pathological loading. A study by Reurink et al. on 53 athletes with hamstring injury showed high-signal intensity changes in 89% of the participants and 42% showed low-signal intensity changes in MRI after return to play, which was after an average of 28 days [36]. In contrast, we observed hardly any signal intensity changes in our long-term follow-up cohort, leading to the assumption that initially seen muscle edema and formation of fibrous tissue declines over time. In terms of muscular atrophy of the hamstring muscles, 23/25 patients (92%) had fatty muscle infiltration of Goutallier Grade 0 or 1 which can be considered as non-pathological [14]. Two patients (8%) demonstrated Grade 2 and Grade 3 fatty infiltration on the operated side which appears to be irreversible after a certain stage and is associated with decreased muscle function [30, 43]. In our study, patients demonstrated significant differences in fatty infiltration between the injured and uninjured limb and radiological volumetry revealed a mean recovered hamstring volume of 94%. The interlimb volume asymmetry is classified within the accepted 10% benchmark. These findings are consistent with the results of the study by Pihl et al., where a mean muscle volume deficit of 9%, and significantly greater total fatty infiltration in the injured hamstring muscle group is described [34]. Additionally, our study confirms the reported correlation among deficits in muscle strength and hamstring volume [34]. We found a moderate correlation among interlimb asymmetries in muscle strength and muscle volume and a strong significant correlation when only patients with their operated limb inferior to their healthy limb are considered. Based on these results, we may, to a certain extent, infer adequate muscle volume recovery from high muscle strength recovery and thereby forego an MRI examination and achieve a cost-effective postoperative rehabilitation monitoring, which can be implemented in daily practice. Nevertheless, subjective clinical outcome should be considered separately from outcomes concerning muscle strength, fatty infiltration, and hamstring volume, as these do not correlate with each other. Overall patient outcome cannot be derived solely from the subjective clinical outcome on the one hand or the strength and MRI results on the other. To obtain an overall outcome, a combination of subjective clinical outcome paired with strength testing, from which conclusions can then be drawn about the volume, is the most sensible and cost-efficient approach.

Although patients achieve satisfactory recovery of hamstring strength and volume, interlimb asymmetries persist and are not reflected in the clinical scores. This could be due to the higher age of the patients and their lower level of activity. Remaining deficits in hamstring strength and volume might only be revealed at higher activity levels (e.g., young age and athletes) and may not be adequately captured by current evaluation scores. Therefore, more sensitive hamstring- and sport-specific scoring methods for more athletically active collectives are needed to adequately assess postoperative functional status. The persisting interlimb asymmetries could also result from inadequately performed postoperative rehabilitation. Greater emphasis should be placed on early functional aftercare and targeted hamstring strength and muscle building to reduce remaining asymmetries.

This study is the first to assess correlation of subjective clinical outcome with postoperative hamstring strength and radiological outcome in terms of fatty infiltration and muscle volume after proximal hamstring tendon repair. Compared to previous studies, a relatively large cohort was acquired, and follow-up protocol could be carried out in one day. Several of the most frequently used clinical variables of prior studies were utilized in this study, enabling an effective comparison. Nevertheless, we need to handle the results cautiously, as there are certain limitations to this study. First, our conducted study is retrospective and lacked a control group of conservatively treated patients, so no comparison was possible, limiting our results. Next, we should be careful when evaluating functional and radiological results, as there is no preoperative data to compare our results to and there may have been relevant strength and volume asymmetries beforehand. Furthermore, the Goutallier score is not validated for the hamstring muscles and is only a visual impression of an image. Regarding high rates of function, it should be taken in account that our cohort had a mean age of 51 years, and their activity level and physical demand is lower compared to younger patients. It is likely that the remaining strength and volume deficits become more restrictive in a younger, more active population and therefore the clinical outcome may differ. Conversely, restoring pre-injury strength depends on and becomes more difficult with age, enabling younger athletes to more easily compensate interlimb asymmetries. The patients lost to follow-up strength testing and imaging, as well as the different timings of follow-up bias the outcome of the study. Strength and imaging findings may differ significantly between a 12- or 76-months follow-up.

## Conclusion

Proximal hamstring tendon repair leads to satisfactory results regarding clinical outcome with fully integrated muscle–tendon unit at mean 3.5 years following surgery. Patients show good recovery of hamstring strength and volume in the injured limb. Based on the correlation between asymmetry in hamstring strength and volume, it may be sufficient to assess hamstring strength asymmetry and from it infer muscle volume asymmetry, eliminating the strict need for an MRI scan at future postoperative follow-up examinations. Interlimb asymmetries, in terms of muscle strength, fatty infiltration, and hamstring volume do not correlate with clinical outcome and should always be determined and interpreted separately.

## Data Availability

The data that support the findings of this study are available upon reasonable request from the corresponding author. Restrictions apply to the availability of some datasets, which are not publicly accessible due to [ethical/legal/privacy reasons].

## Abbreviations

HRQOL-4: Health related quality of life—Healthy Days Core Module
NRS: Numeric rating scale
UHD: Unhealthy days index
SSS: Simple summary score
mHHS: Modified Harris Hip Score
TAS: Tegner Activity Scale
RTPA: Return to pre-injury activity level
MRI: Magnetic resonance imaging
LSI: Limb symmetry index
KG: Kilogram
MV: Muscle volume
TIRM: Turbo-inversion recovery-magnitude
TR: Repetition time
TE: Time to echo
ST: Slice thickness
FOV: Field of view
PD: Proton density
TSE: Turbo spin echo
ROI: Regions of interest
ST: Semitendinosus
SM: Semimembranosus
BF: Biceps femoris
CT: Computed tomography
SD: Standard deviation
